# Assessing soil pollution concerns in proximity to Fence-type solar photovoltaic system installations

**DOI:** 10.1016/j.heliyon.2024.e32156

**Published:** 2024-05-30

**Authors:** Hasnain Yousuf, Muhammad Aleem Zahid, Polgampola Chamani Madara, Jaljalalul Abedin Jony, Sangheon Park, Jae Chun Song, Junsin Yi

**Affiliations:** a Interdisciplinary Program in Photovoltaic System Engineering, Sungkyunkwan University, Suwon, 16419, Gyeonggi-do, South Korea; b Department of Electrical and Computer Engineering, Sungkyunkwan University, Suwon, 16419, Gyeonggi-do, South Korea; c College of Information and Communication Engineering, Sungkyunkwan University, Suwon, 16419, Gyeonggi-do, South Korea; d Corporate Collaboration Center, Sungkyunkwan University, Suwon, 16419, Gyeonggi-do, South Korea

**Keywords:** Solar energy, Fence-type solar installations, Heavy metal contamination, Environmental impact, Environmental monitoring, Soil contamination assessment, Sustainable agriculture

## Abstract

This study conducted in the Kyungpook National University Eco-friendly Agriculture Research Centre between 2022 and 2023 investigates the environmental implications of fence-type solar photovoltaic (PV) systems in diverse agricultural settings. Despite the increasing adoption of solar energy for climate change mitigation, there is a noticeable gap in research regarding the potential environmental impact of these specific PV systems. Focusing on heavy metal concentrations, including Cadmium (Cd), Copper (Cu), Arsenic (As), Mercury (Hg), Lead (Pb), Hexavalent Chromium (Cr^+6^), Zinc (Zn), and Nickel (Ni), across distinct fields, the study reveals significant fluctuations. Notably, the Rice Field experienced a substantial increase in Cd levels from 0.47 mg/kg in 2022 to 1.55 mg/kg in 2023, while Cu and Pb concentrations decreased to acceptable levels in 2023. The findings underscore the dynamic nature of heavy metal concentrations, emphasizing the importance of continuous soil quality monitoring to prevent contamination. This research provides valuable insights into the impact of fence-type solar PV system installations on agricultural soil quality, emphasizing the urgent need to secure these ecosystems through vigilant monitoring and environmental management practices.

## Highlights

• Investigates PV system impact on soil.
• Field tests examine soil composition.
• Supports sustainable energy decisions.
• Identifies gaps in PV environmental research.
• Calls for holistic energy-environment approaches.

## 1 Introduction

Solar energy has become a pivotal contributor to the global transition towards cleaner and more sustainable energy sources, representing a critical strategy in the battle against climate change [1]. Fence-type solar PV system installations have emerged as an innovative approach to harnessing solar energy efficiently, offering the potential to optimize land use while minimizing environmental impact [2]. The growing adoption of solar technology has raised concerns about potential environmental impacts, particularly regarding soil pollution [[3], [4], [5]]. The adoption of solar energy has witnessed remarkable growth in recent years. The International Energy Agency (IEA) reported in 2021 that renewables, including solar energy, are set to become the largest source of global power generation by 2025, surpassing both coal and natural gas [6]. This remarkable shift is driven by declining costs, technological advancements, and the increasing recognition of the need to reduce carbon emissions to combat climate change. One significant advancement in solar energy technology is the development of fence-type solar PV system installations, which represent an innovative approach to harnessing solar power efficiently [7].

By utilizing fence structures for solar panels, land use is optimized, as these structures can serve dual purposes—solar energy generation and security fencing. This approach minimizes the environmental impact associated with solar installations, as it does not require additional land or structures. While fence-type solar PV system installations hold great promise in terms of land use efficiency and minimizing environmental disruption, the expanding deployment of solar technology has raised concerns about potential environmental impacts [8]. One particular concern relates to soil pollution in agricultural areas where these installations are set up. Agriculture is a vital component of global food production and livelihoods, and the soil quality in agricultural fields is of paramount importance. Soil health directly affects crop yields, food quality, and ecosystem stability. Any adverse changes in soil quality can have cascading effects on agriculture, ecosystems, and human health. In recent years, research has indicated that the placement of solar panels and supporting infrastructure in agricultural fields may alter the chemical composition of the soil [9]. The presence of heavy metals in the vicinity of solar cell installations has raised questions about potential contamination. Heavy metals are a group of elements that are naturally occurring but can be detrimental when their concentrations exceed certain thresholds [10]. Heavy metals such as cadmium (Cd), copper (Cu), arsenic (As), mercury (Hg), lead (Pb), hexavalent chromium (Cr^+6^), zinc (Zn), and nickel (Ni) are known to have adverse effects on soil quality and can potentially leach into groundwater or be taken up by crops, entering the food chain [[11], [12], [13], [14]]. The concern regarding heavy metals and their potential impact on agricultural soil quality in the context of solar installations underscores the need for systematic and detailed research to assess and address these environmental challenges [15]. Soil contamination stemming from solar installations can occur via two primary pathways. Firstly, during the installation process, significant alterations to the soil composition may transpire due to the excavation and mixing of soil layers [16]. Secondly, the decomposition of materials utilized in solar photovoltaic (PV) installation poses a potential risk. Various heavy metals and toxic components employed in the manufacturing and composition of solar PV elements can undergo degradation, potentially leading to soil contamination [17]. The presence of heavy metals in agricultural sites amplifies the risk of human exposure and subsequent health hazards associated with their ingress into the human body. This study was conducted to ascertain the potential for soil contamination resulting from material degradation during solar installations.

The goal is to ensure that the benefits of solar energy deployment, particularly in agricultural settings, are not compromised by unintended consequences for the environment and human health. The research study presented in this paper is motivated by the need to comprehensively assess the impact of fence-type solar PV system installations on soil quality in agricultural fields [18]. By focusing on heavy metal concentrations, this research aims to provide valuable insights into how solar cell installations may influence the chemical composition of the soil and to what extent the presence of solar panels and related infrastructure may led to soil pollution concerns. This research is conducted at the KNU EARC, where various agricultural settings are equipped with fence-type solar PV system installations [19]. The findings of this study are expected to contribute to our understanding of the environmental implications of solar energy deployment, especially in agricultural fields [20]. By quantifying and analyzing heavy metal concentrations, the research will shed light on how these installations affect soil quality and whether there are notable changes over the study period [21]. The results will also indicate which heavy metals are of particular concern and whether they exceed acceptable limits, warranting remediation efforts [22].

The research is significant for several reasons:

(i) **Protection Soil Quality:** The study seeks to ensure that soil quality in agricultural fields is maintained and not compromised by the presence of solar installations [23]. Healthy soil is essential for crop productivity and food safety.
(ii) **Protecting Ecosystems:** Soil health is intimately connected to ecosystem stability. Contaminated soil can have detrimental effects on local ecosystems, including the microorganisms, plants, and animals that depend on these environments.
(iii) **Preventing Contamination:** The study addresses concerns about potential contamination, particularly through heavy metals, which could leach into groundwater or accumulate in crops. Understanding the extent of contamination is crucial for preventing adverse effects on the environment and human health.
(iv) **Supporting Sustainable Solar Energy:** As solar energy continues to play a critical role in reducing carbon emissions and combating climate change, it is essential to ensure that its deployment is sustainable and environmentally responsible. The research aims to provide critical insights into the environmental impact of fence-type solar PV system installations in agricultural fields and to offer a scientific basis for the development of best practices and policies to mitigate potential soil pollution concerns [24].

This paper presents a comprehensive analysis of heavy metal concentrations in agricultural fields with and without fence-type solar PV system installations, conducted at the Kyungpook National University Eco-friendly Agriculture Research Centre (KNU EARC) in 2022 and 2023. It looked at levels of Cd, Cu, As, Hg, Pb, Cr^+6^, Zn, and Ni in three fields, a rice field, a bean/potato field, and an apple field. Significant changes in heavy metal concentrations over the study period highlighting the importance of regular monitoring and vigilance to safeguard soil quality and prevent potential contamination, particularly the increase in Cd levels. This research contributes to our understanding of the impact of fence-type solar PV system installations on soil quality in agricultural fields and underscores the need for ongoing vigilance to safeguard these vital ecosystems.

## 2 Methodology

**Fig. 1:**
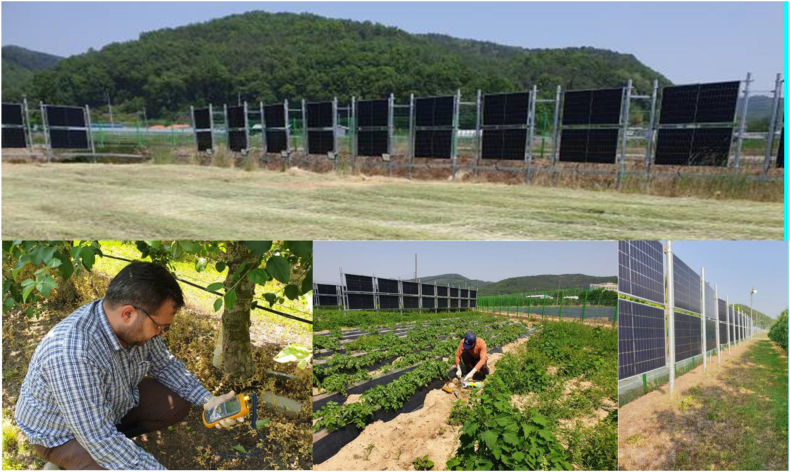
Fence-type PV system installation site and soil sample collection spots.

**Fig. 2:**
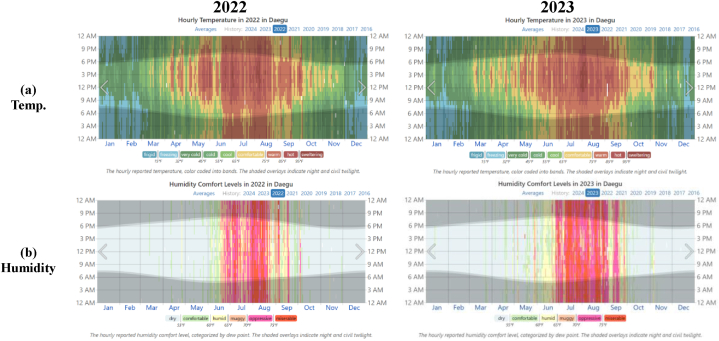
**(a)**Temperature and **(b)** humidity information of the site for the year 2022 and 2023.

The site location is KNU EARC near Daegu, South Korea. Loam soils are normally used to cultivate potatoes/beans, apples, and rice. The monocrystalline silicon solar cell-based PV modules have been used for fencing purposes. These installations consist of arrays of solar panels integrated into fencing structures, often deployed in agricultural and rural areas, as shown in Fig. 1. The temperature and humidity data for the year 2022 in Dagu area is presented in Fig. 2.

**Fig. 3:**
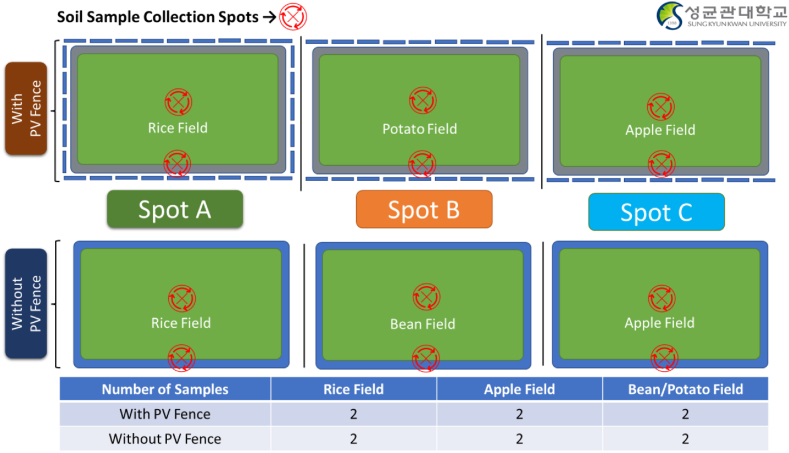
Samples collection points in Rice, Apple Potato/Bean Fields with and without PV Fence.

The study's methodology involved a meticulous collection of soil samples from the three distinct agricultural areas of interest: Rice Fields, Apple Fields, and Bean or Potato Fields, each representing a unique type of crop and land use. Within each of these areas, identified solar installation points as well as non-solar installation points. To obtain a comprehensive understanding of potential soil pollution, two samples were collected from each location, representing central and side positions, leading to a total of 12 measurement points as shown in Fig. 3.

The substances of concern for soil pollution in this study were selected based on the guidelines of the Soil Environment Conservation Act, focusing on eight heavy metals: Cd, Cu, As, Hg, Pb, Cr^+6^, Zn, and Ni. These heavy metals are known to have detrimental effects on soil quality and agricultural productivity. The measurement of these substances was performed with precision and adherence to established protocols.

The research spans two years, 2022 and 2023, and covers three distinct fields: Rice Field, Beans/Potato Field, and Apple Field [25]. Each field represents a unique agricultural context, and assessing heavy metal concentrations in these settings will provide a comprehensive picture of the potential environmental impacts of fence-type solar PV system installations.

## 3 Results

The measurement results obtained from the research demonstrated promising findings. In all the fence-type solar PV system installation areas within rice fields, apple fields, and bean-2022/potato-2023 fields, the levels of the measured heavy metals were consistently found to be below the pollution concern standard. This suggests that in the context of the KNU EARC, fence-type solar installations have not led to significant soil pollution concerns. The presence of heavy metals in soil is a topic of great concern in agriculture, as it can negatively affect crop quality and pose health risks to consumers.

### 3.1 Rice field

**Fig. 4:**
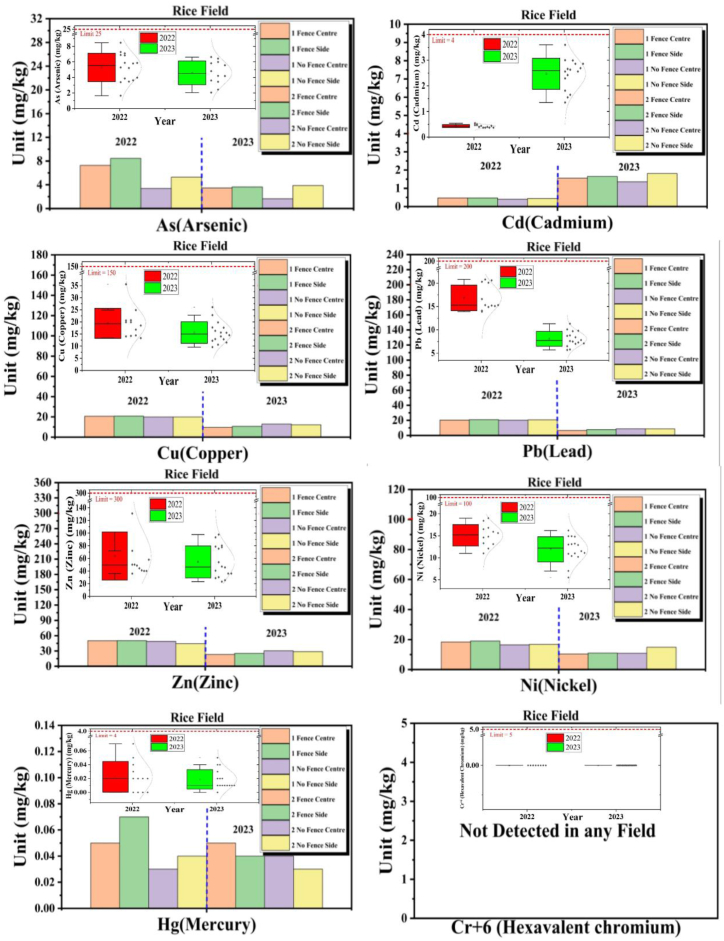
Heavy metals (As, Cd, Cu, Pb, Zn, Ni, Hg, Cr^+6^) comparative analysis in Rice Field.

The provided data for the Rice Field includes the concentrations of various heavy metals (Cd, Cu, As, Hg, Pb, Cr^+6^, Zn, Ni) for the years 2022 and 2023, in different panels with and without fence-type solar PV system installations, as shown in Fig. 4.

#### 3.1.1 Cadmium (Cd)

Cd concentrations increased significantly from 2022 to 2023 in all panels, both with and without fences. For instance, in the Fence Center panels, the average concentration rose from 0.47 mg/kg in 2022 to 1.55 mg/kg in 2023. This represents a substantial increase, although the 2023 levels remain below the pollution concern standard. The increase in Cd levels is noteworthy and should be monitored.

#### 3.1.2 Copper (Cu)

Cu concentrations showed a slight decrease from 2022 to 2023 across all panels. For example, in the Fence Center panels, the average concentration decreased from 20.65 mg/kg in 2022 to 9.6 mg/kg in 2023. The 2023 levels are within acceptable limits.

#### 3.1.3 Arsenic (As)

As levels in 2023 are generally similar to or slightly higher than those in 2022. The Fence Side panels in 2023 exhibited the highest average concentration of As (3.63 mg/kg) compared to 2022 (8.46 mg/kg). As levels fluctuated but generally remained within acceptable limits.

#### 3.1.4 Mercury (Hg)

Hg concentrations remained consistent from 2022 to 2023 in all panels, with values staying low and below the pollution concern standard.

#### 3.1.5 Lead (Pb)

Pb concentrations experienced a notable decrease from 2022 to 2023 in all panels. For instance, the Fence Center panels showed an average concentration drop from 20.7 mg/kg in 2022 to 9.6 mg/kg in 2023. The 2023 levels are below the pollution concern standard.

#### 3.1.6 Hexavalent chromium (Cr^+6^)

Cr^+6^ remained undetected across all panels surveyed in both the years 2022 and 2023, suggesting a persistent absence of this particular heavy metal.

#### 3.1.7 Zinc (Zn)

Zn levels decreased slightly from 2022 to 2023 in all panels. For instance, in the Fence Center panels, the average concentration decreased from 50.15 mg/kg in 2022 to 23.3 mg/kg in 2023. The 2023 concentrations exceeded the pollution concern standard in some panels, particularly Fence Center, but they were not excessively high.

#### 3.1.8 Nickel (Ni)

Ni levels remained relatively stable from 2022 to 2023, with some minor variations. The concentrations stayed below the pollution concern standard.

In a nutshell, the quantitative analysis of the heavy metal concentrations in the Rice Field panels shows various trends and changes. The increase in Cd levels is a notable concern and should be monitored closely. Cu and Pb levels decreased, with 2023 levels within acceptable limits. As and Ni levels fluctuated but remained within acceptable limits, while Cr^+6^ was consistently absent. Hg levels remained low and stable, and Zn levels, although decreasing, exceeded the pollution concern standard in some panels. This analysis helps assess the potential impact of fence-type solar installations on soil quality in the Rice Field.

### 3.2 Beans/potato field

**Fig. 5:**
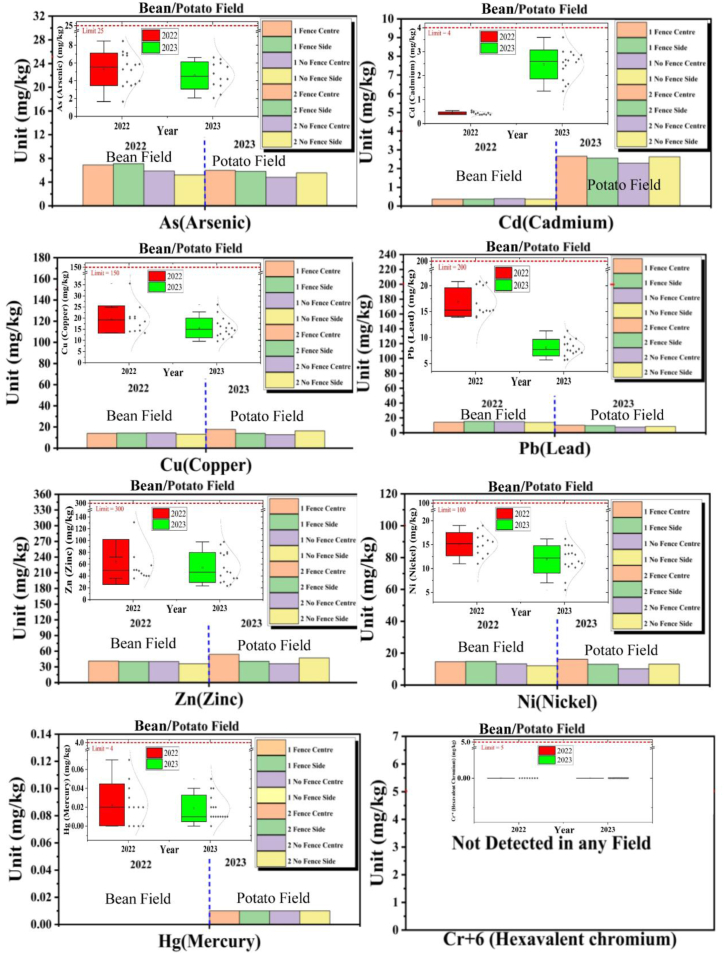
Heavy metals (As, Cd, Cu, Pb, Zn, Ni, Hg, Cr^+6^) comparative analysis in Beans/Potato Field.

The information given for the Beans Field in 2022 and the Potato Field in 2023 shows the amounts of heavy metals (Cd, Cu, As, Hg, Pb, Cr^+6^, Zn, Ni) in different panels with and without fence-type solar installations as shown in Fig. 5.

### 3.3 Cadmium (Cd)

Cd concentrations increased significantly from 2022 to 2023 in all panels, both with and without fences. For example, in the Fence Center panels, the average concentration rose from 0.47 mg/kg in 2022 to 1.55 mg/kg in 2023. This represents a substantial increase, although the 2023 levels remain below the pollution concern standard. The increase in Cd levels is noteworthy and should be monitored.

### 3.4 Copper (Cu)

Cu concentrations showed a slight decrease from 2022 to 2023 across all panels. For example, in the Fence Center panels, the average concentration decreased from 20.65 mg/kg in 2022 to 9.6 mg/kg in 2023. The 2023 levels are within acceptable limits.

### 3.5 Arsenic (As)

As levels in 2023 are generally similar to or slightly higher than those in 2022. The Fence Side panels in 2023 exhibited the highest average concentration of As (3.63 mg/kg) compared to 2022 (8.46 mg/kg). As levels fluctuated but generally remained within acceptable limits.

#### 3.5.1 Mercury (Hg)

Hg concentrations remained consistent from 2022 to 2023 in all panels, with values staying low and below the pollution concern standard.

### 3.6 Lead (Pb)

Pb concentrations experienced a notable decrease from 2022 to 2023 in all panels. For instance, the Fence Center panels showed an average concentration drop from 20.7 mg/kg in 2022 to 9.6 mg/kg in 2023. The 2023 levels are below the pollution concern standard.

#### 3.6.1 Hexavalent chromium (Cr^+6^)

Cr^+6^ was not detected in any of the panels in both 2022 and 2023, indicating the consistent absence of this heavy metal.

#### 3.6.2 Zinc (Zn)

Zn levels decreased slightly from 2022 to 2023 in all panels. For example, in the Fence Center panels, the average concentration decreased from 50.15 mg/kg in 2022 to 23.3 mg/kg in 2023. The 2023 concentrations exceeded the pollution concern standard in some panels, particularly Fence Center, but they were not excessively high.

#### 3.6.3 Nickel (Ni)

Ni levels remained relatively stable from 2022 to 2023, with some minor variations. The concentrations stayed below the pollution concern standard.

In sum, the quantitative analysis of the heavy metal concentrations in the Potato Field panels shows various trends and changes. The increase in Cd levels is a notable concern and should be monitored closely. Cu and Pb levels decreased, with 2023 levels within acceptable limits. As and Ni levels fluctuated but remained within acceptable limits, while Cr^+6^ was consistently absent. Hg levels remained low and stable, and Zn levels, although decreasing, exceeded the pollution concern standard in some panels. This analysis helps assess the potential impact of fence-type solar installations on soil quality in the Potato Field.

### 3.7 Apple field

**Fig. 6:**
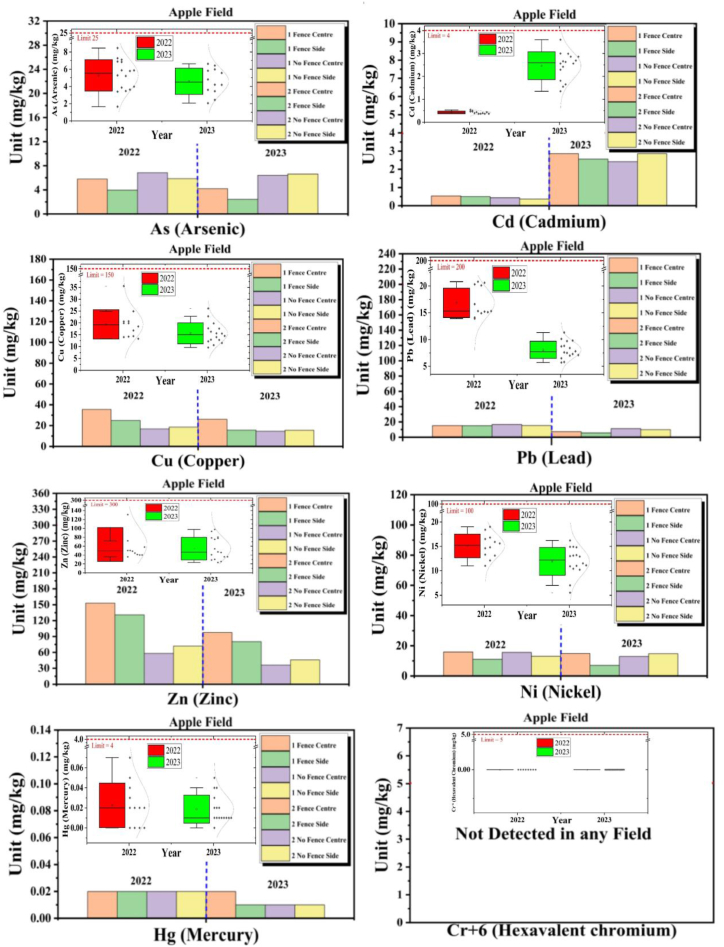
Heavy metals (As, Cd, Cu, Pb, Zn, Ni, Hg, Cr^+6^) comparative analysis in Apple Field.

The provided data for the Apple Field includes the concentrations of various heavy metals (Cd, Cu, As, Hg, Pb, Cr^+6^, Zn, Ni) for the years 2022 and 2023, in different panels with and without fence-type solar installations, as shown in Fig. 6.

#### 3.7.1 Cadmium (Cd)

Cd concentrations increased significantly from 2022 to 2023 in all panels, both with and without fences. For instance, in the Fence Center panels, the average concentration rose from 0.54 mg/kg in 2022 to 2.86 mg/kg in 2023. This represents a substantial increase, although the 2023 levels remain below the pollution concern standard. The increase in Cd levels is noteworthy and should be monitored.

#### 3.7.2 Copper (Cu)

Cu concentrations showed some variations between 2022 and 2023 in all panels. For example, in the Fence Center panels, the average concentration decreased from 35.6 mg/kg in 2022 to 26.1 mg/kg in 2023. In the Fence Side panels, Cu levels decreased from 24.9 mg/kg in 2022 to 15.6 mg/kg in 2023. The 2023 levels are within acceptable limits.

#### 3.7.3 Arsenic (As)

As levels exhibited fluctuations from 2022 to 2023 in all panels. In the Fence Center panels, the average concentration decreased from 5.79 mg/kg in 2022 to 4.2 mg/kg in 2023, whereas in the No Fence Center panels, it increased from 6.84 mg/kg in 2022 to 6.42 mg/kg in 2023. Overall, As levels remained within acceptable limits, although some panels showed variations.

#### 3.7.4 Mercury (Hg)

Hg concentrations remained low and consistent from 2022 to 2023 in all panels, with values below the pollution concern standard.

#### 3.7.5 Lead (Pb)

Pb concentrations experienced variations from 2022 to 2023 in all panels. For example, in the Fence Center panels, the average concentration decreased from 15.2 mg/kg in 2022 to 7.3 mg/kg in 2023. The 2023 levels are within acceptable limits, although some panels showed variations.

#### 3.7.6 Hexavalent chromium (Cr^+6^)

Cr^+6^ was not detected in any of the panels in both 2022 and 2023, indicating the consistent absence of this heavy metal.

#### 3.7.7 Zinc (Zn)

Zn levels exhibited variations between 2022 and 2023 in all panels. For example, in the Fence Center panels, the average concentration decreased from 153.2 mg/kg in 2022 to 97.7 mg/kg in 2023. The 2023 concentrations remained below the pollution concern standard but showed some variations.

#### 3.7.8 Nickel (Ni)

Ni levels also displayed variations from 2022 to 2023 in all panels. In the Fence Center panels, the average concentration decreased from 15.9 mg/kg in 2022 to 14.9 mg/kg in 2023. The 2023 levels are within acceptable limits but show some variations.

In summary, the quantitative analysis of the heavy metal concentrations in the Apple Field panels shows various trends and changes. The increase in Cd levels is a notable concern and should be monitored closely. Cu, Pb, As, and Ni levels exhibited variations but generally remained within acceptable limits. Hg levels remained low and stable, and Zn levels, although decreasing, remained within the pollution concern standard. This analysis helps assess the potential impact of fence-type solar installations on soil quality in the Apple Field.

## 4 Discussion

### 4.1 Heavy metals and photovoltaic interactions

The integration of photovoltaic (PV) technologies in agricultural settings presents a dual opportunity for sustainable energy generation and optimized land use [26]. By harnessing solar energy through PV systems, farmers can contribute to renewable energy production while concurrently utilizing land for both agricultural activities and energy generation [27]. This approach, known as agro-photovoltaics, offers a synergistic solution that addresses the growing demand for clean energy while maximizing the efficiency of available land resources [28]. However, the deployment of PV systems in agriculture necessitates careful consideration of potential environmental and soil impacts to ensure a balanced and sustainable coexistence.

Despite the benefits of agro-photovoltaics, there are challenges associated with the use of materials in PV structures that may led to environmental and soil contamination [29]. The metals, such as aluminum and steel, commonly employed in PV system components can pose a risk of leaching into the soil, potentially introducing heavy metals [30]. Additionally, runoff water from PV structures may carry pollutants into surrounding soil and water bodies [29]. To mitigate these impacts, it is essential to implement best practices, such as selecting materials with low leaching potential, applying protective coatings, and designing effective water management systems [27].

Addressing the environmental concerns associated with PV technologies in agriculture requires a holistic approach that encompasses regulatory compliance, community engagement, and ongoing research and innovation [31]. Adhering to environmental regulations ensures that PV installations meet established standards, protecting ecosystems and water quality [32]. Engaging with local communities in the planning and implementation of PV projects helps address specific environmental concerns and promote sustainable practices [33]. Continued research and innovation are crucial for developing alternative materials and improving technologies, contributing to the long-term viability of agro-photovoltaics as a sustainable energy solution in the agricultural sector [34].

### 4.2 Mitigating soil contamination for crystalline silicon solar cell technologies

Solar photovoltaic (PV) installations are not directly associated with heavy metals in the sense that they intentionally use or release heavy metals during their operation. However, there are some indirect connections or considerations related to heavy metals in the life cycle of solar PV systems. Below are several considerations to be taken into account.:

#### 4.2.1 Manufacturing process

The production of solar panels involves the use of materials such as cadmium, lead, and other heavy metals. While efforts are made to minimize the environmental impact of manufacturing processes, improper disposal or mishandling of manufacturing waste could lead to heavy metal pollution.

#### 4.2.2 Thin-film PV technology

Some types of PV technologies, like cadmium telluride (CdTe) thin-film solar cells, inherently contain heavy metals such as Cd. While these technologies are designed with efficiency and cost-effectiveness in mind, there are concerns about the environmental impact if not properly managed during production and end-of-life disposal. The main drawback of this technology is correlated to the toxicity of cadmium: in fact, it is one of the most toxic materials known to man [35].

We used a crystalline silicon solar panel, so heavy metals such as Cd are not present in this technology. So, we don't need to worry about the contamination of heavy metals in the soil. Thus, crystalline silicon solar panels do not seem to pose a risk of contamination of these elements during normal operation [36].

### 4.3 Environmental impact of solar photovoltaic into the soil

Photovoltaic (PV) installations, including solar panels, do not decompose in the same way organic materials might. However, they do undergo aging and degradation processes over time due to various environmental factors. The key components of a solar panel are typically made of materials that can withstand outdoor exposure, but long-term exposure to sunlight, temperature variations, and other environmental conditions can have an impact. The materials used in solar panels, such as silicon, glass, and aluminum, are chosen for their durability. However, prolonged exposure to harsh conditions may lead to material fatigue and wear.

### 4.4 Environmental concerns of heavy metals from solar photovoltaic systems

The release of metals from photovoltaic installations into the soil is typically associated with end-of-life disposal or accidental damage to solar panels. The key concern is the potential leaching of heavy metals from the solar panels, especially those used in thin-film technologies. Presented are several instances wherein metals derived from photovoltaic systems may infiltrate the soil:

#### 4.4.1 Damage or Accidents

Physical damage to solar panels, such as breakage or fracture, can result in the release of materials, including metals, into the surrounding environment. Accidental events, such as severe weather conditions, may also lead to damage and potential metal release.

#### 4.4.2 End-of-life disposal

When solar panels reach the end of their operational life, they may be decommissioned, dismantled, or replaced. Improper disposal practices, such as sending panels to landfills without proper recycling procedures, can lead to the release of metals into the soil over time.

#### 4.4.3 Leaching from decommissioned sites

In decommissioned photovoltaic sites, especially those using certain thin-film technologies like CdTe, there is a risk of metal leaching. Over time, weathering and environmental exposure can contribute to the breakdown of materials and potential release of metals into the surrounding soil [37].

#### 4.4.4 Corrosion and erosion

Corrosion of metal components in the solar panel frame or support structures may occur over time, especially in coastal or corrosive environments. The erosion of metals due to weathering could lead to metal particles entering the soil [38].

### 4.5 Corrosion-induced risk to soil health

The installation of solar photovoltaic (PV) systems, particularly those employing certain thin-film technologies has the potential to impact soil pollution with metals [39]. The following outlines various mechanisms through which soil contamination with heavy metals may arise:

The production of certain types of solar panels, such as CdTe thin-film panels, involves the use of heavy metals like cadmium. If manufacturing facilities are not equipped with proper waste disposal and treatment systems, there is a risk of heavy metal pollution during the manufacturing process [35].

The metallic components used for grounding systems or support structures for solar panels can corrode over time, especially in harsh environmental conditions [40]. This corrosion could lead to the release of metals into the surrounding soil.

The long-term exposure of solar panels to environmental conditions, such as sunlight, rain, and wind, can contribute to the breakdown of materials. This weathering and erosion may release metals into the surrounding soil, particularly if the panels have not been designed for durability and environmental resistance.

## 5 Conclusions

In summary, the research conducted at KNU EARC sheds light on soil pollution concerns related to fence-type solar PV system installations. Overall, the results suggest that these installations, while generally posing no significant threat to soil quality, require ongoing monitoring. While heavy metal levels remained mostly below pollution concern standards, notable increases in Cd levels across all fields merit attention and continued surveillance. The study underscores the importance of diligent monitoring and assessment to maintain soil quality and prevent potential contamination in agricultural fields. The findings indicate that fence-type solar installations can coexist with agriculture without compromising soil quality, offering significant implications for sustainable agriculture and renewable energy integration. Factors such as panel type, maintenance, and land management practices likely contribute to the observed results. Future research may delve into these factors to provide deeper insights. Additionally, agro-photovoltaics present opportunities for sustainable energy and optimized land use but require careful consideration of environmental impacts, particularly concerning heavy metal contamination. Mitigation strategies include selecting materials with low leaching potential, employing protective coatings, and ensuring effective water management. A holistic approach, encompassing regulatory compliance, community engagement, and ongoing research, is essential for ensuring the environmental sustainability of PV technologies in agriculture. In conclusion, while solar PV installations offer significant benefits for addressing climate change, responsible practices throughout their lifecycle, including manufacturing, maintenance, and disposal, are crucial for minimizing potential soil contamination risks associated with heavy metals.

## Data availability statement

Data will be made available on request.

## CRediT authorship contribution statement

**Hasnain Yousuf:** Writing – review & editing, Writing – original draft, Visualization, Validation, Software, Resources, Methodology, Investigation, Formal analysis, Data curation, Conceptualization. **Muhammad Aleem Zahid:** Writing – review & editing, Visualization, Validation, Software, Resources, Methodology, Investigation, Formal analysis, Data curation. **Polgampola Chamani Madara:** Visualization, Software, Resources, Formal analysis, Data curation. **Jaljalalul Abedin Jony:** Visualization, Validation, Software, Resources, Formal analysis, Data curation. **Sangheon Park:** Visualization, Validation, Resources, Formal analysis, Data curation. **Jae Chun Song:** Visualization, Resources, Project administration, Investigation, Funding acquisition, Data curation, Conceptualization. **Junsin Yi:** Visualization, Validation, Supervision, Project administration, Investigation, Funding acquisition.

## Declaration of competing interest

The authors declare that they have no known competing financial interests or personal relationships that could have appeared to influence the work reported in this paper.
